# Direct determination of amorphous number density from the reduced pair distribution function

**DOI:** 10.1016/j.mex.2019.03.005

**Published:** 2019-03-21

**Authors:** Georgios S.E. Antipas, Konstantinos T. Karalis

**Affiliations:** a Molecular Modelling Laboratory, Park Innovaare, 5234 Villigen PSI, Switzerland; b Laboratory for Scientific Computing and Modelling, Paul Scherrer Institute, 5232 Villigen PSI, Switzerland

**Keywords:** PDFD: PDF (number) density, Amorphous, Density, Amorphous number density, Reduced pair distribution function, Diffraction

## Abstract

The inference of amorphous bulk density, while straightforward for nonporous, soluble materials, may present a formidable challenge in some of the most important classes of industrial applications, involving melts, porous solids, and non-soluble organic pharmaceuticals, with varied implications depending on the material’s level of technological interest. Within nanotechnology and the life sciences in particular, accurate determination of amorphous true density is a frequent requirement and a regular puzzle, when, e.g., neither the Archimedean principle nor gas pycnometry may be applied, the former being only applicable to insoluble compounds, while the latter yielding skeletal density – an overestimate of true density to the extent of blind pores – and its efficiency is affected by the choice of the gas medium. In these cases, it is feasible to infer amorphous density from diffraction experiments through the use of the reduced Pair Distribution Function (PDF). Although an estimate of crystalline density has been known to be possible via the PDF shape, here we outline a new method extending this facility to include the estimation of amorphous density.

• Amorphous density may be inferred from the position of a local minimum of the reduced PDF profile, the latter extracted via a Fourier transformation of collected diffraction intensity.
• The PDF minimum is located within the PDF range bounded by r_min_ = 2π/Q_max_ and the position of the first coordination peak, where Q_max_ is the maximum length of the scattering vector achieved in the diffraction experiment.
• Amorphous density is calculated as the ratio of the value of the reduced PDF at the local minimum, divided by the term 4πr, where r is the real space coordinate of the local minimum.

## Graphical abstract

**Figure:**
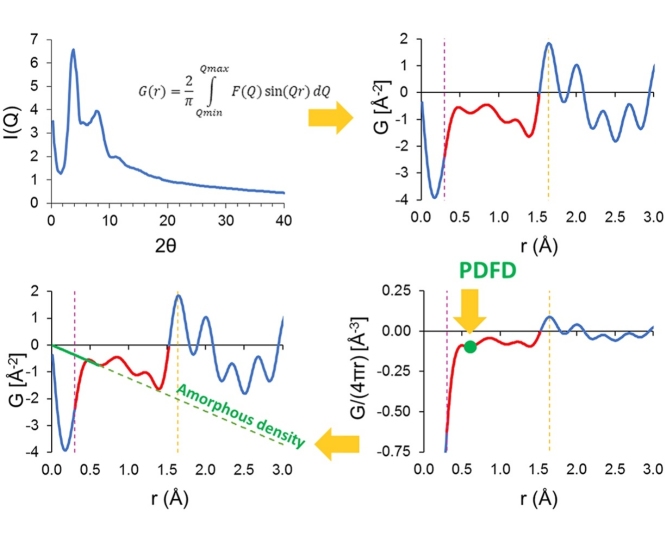

Specifications Table

**Table:** 

**Subject area:**	Materials Science
**More specific subject area:**	Computational Materials Science
**Method name:**	PDFD: PDF (number) density
**Name and reference of original method:**	*Not applicable*
**Resource availability:**	*Supplementary Material*

## Method details

In the case of perfectly periodic materials, it is possible to utilize pair correlation statistics in order to estimate the material’s bulk number density, ρ_0_. For these materials, the reduced PDF, symbolized as G(r), is the sum of peak information and of a straight sloping baseline expressed as −4πρ_0_r and intrinsically feasible to work out from the PDF shape. However, upon increasing deviation from bulk crystallinity, this baseline becomes affected by the dominating presence of an attenuating particle shape factor, γ(r), and is, then, expressed as −4πρ_0_γ(r)r [1]. The extreme case of linearity deviation towards high values of the real space coordinate, r, is that of completely amorphous materials [[2], [3], [4], [5], [6], [7]], in which case ρ_0_ may not be estimated from the tangent of the baseline as the latter is no longer a straight line [8]. In some cases, numerical estimation (i.e. the identification of PDF peaks and, as a result, of what constitutes baseline) has been cited as possible via modelling [1], but in practice this is not readily applicable to the amorphous state. Additionally, although in principle the PDF baseline may be estimated from a Fourier transform of Small Angle Scattering (SAS) data [8], the pair density function, ρ(r), which feeds into the calculation requires use of ab initio structural models, and we are not aware of such paradigms applied to amorphous materials in the literature.

Aiming to provide a reliable alternative for the determination of amorphous density from diffraction intensity datasets, here we propose a simple but functional methodology which relies on a numerical manipulation of the reduced PDF. Following we outline the proposed methodology in two steps.

1 **Collection of diffraction intensity and relation to G(r)**. To derive an approximation of amorphous number density, we employ the fundamental relation between diffraction and the PDF, by first performing a diffraction experiment, correcting the collected spectra for incoherent, multiple and background scattering and then Fourier transforming (FT) the coherent/elastic part of the collected intensity, I(Q), to obtain the reduced PDF, G(r), as

(1) $$G\left(r\right)=\frac{2}{\pi }\int_{Qmin}^{Qmax}F\left(Q\right)\text{sin}\left(Qr\right)dQ$$

where F(Q) is the reduced structure factor, for which F(Q)^2^=I(Q) and F(Q)=Q[S(Q)-1] where S(Q) is the material’s structure factor [3]. Eq. (1) is independent of the radiation source and gives best results primarily for electron diffraction (driven by recent breakthroughs in Transmission Electron Microscopy ePDF analysis of nanoparticles and disordered materials [9]) but also for monochromatic powder X ray diffraction (XRD).

I(Q) is experimentally collected over a range of reciprocal space vectors, Q, the lower and upper limits of which – Q_min_ and Q_max_ respectively – are functions of the limitations set on the range of scattering angles, 2θ, and on the radiation wavelength, λ, by the experimental apparatus, via Q = 4πsin(θ)/λ. To derive physically meaningful PDF curves, the FT in Eq. (1) mandates that Q_max_ is chosen such that F(Q_max_) = 0 (or S(Q_max_) = 1), in order to avoid finite-size effects/artifacts in the low r range. The best practice would then be to compare G(r) shapes calculated across a range of Q_max_ cut-off values, sampling the reduced PDF at real space intervals no larger than the Nyquist rate, equal to π/Q_max_ (oversampling will not cause harm, however), while always seeking to disregard peaks/shoulders which do not consistently appear on all PDF curves under consideration. Additionally, Q_max_ cut-off’s causing noticeable G(r) peak position shifting should be disregarded. The part of G(r) common to all Q_max_ cut-off’s can then be safely considered for use in the next step.

2 **Real space G(r) sampling and numerical manipulation**. PDF importance lies in the pair correlation statistics which connect G(r) to the material’s (bulk) number density, ρ_0_, via the *atomic PDF*, g(r)

(2) $$g\left(r\right)=1+\frac{G\left(r\right)}{4\pi r\rho_{0}}$$

where ρ_0_=N/V, N is the number of atoms in the structure contained in volume V [10]. However, by definition, the atomic PDF is also the ratio of the non-negative pair density function, ρ(r), divided by ρ_0_

(3) $$g\left(r\right)=\rho \left(r\right)/\rho_{0}$$

Hence, ρ(r) may possess a local minimum for which Eq. (3) will be close to zero, and from Eq. (2) it follows that for this local minimum, G(r) ≈ -4πrρ_0_; on the additional provision that G(r) is sampled for r values at least equal to r_min_ = 2π/Q_max_, we may write

(4) $$\left|{\left\{\frac{G\left(r\right)}{4\pi r}\right\}}_{min}^{r\geq r_{min}}\right|=\;\left|\rho \left(r\right)-\rho_{0}\right|\approx \rho_{0}$$

The current methodology is applicable to any amorphous system, as all of its individual steps - i.e. a) collecting diffraction intensity, I(Q), b) correcting the collected intensity dataset depending on the diffraction method (Q_max_), c) Fourier-transforming I(Q) to G(r): via Eq. (1), d) calculating the ratio G(r)/4πr following Eqs. (2), (3), (4) and e) calculating amorphous density from the value of the minimum of G(r)/4πr - are system independent and, as a result, generally applicable. In essence, the current contribution is aimed at making explicit two points of increased merit regarding amorphous G(r); these are:

1 That it is feasible to infer amorphous density via the ratio G(r)/(4πr) and we explain this in the main body of the paper (Eqs. (2), (3), (4)). These equations are system independent, i.e. they are valid for any purely amorphous system, or for amorphous parts of, say, quasicrystalline materials which may be detected – for example – on a TEM.
2 That an estimate of amorphous density may be inferred from the value of the G(r)/(4πr) minimum of the G(r) usable range. However, for our methodology to provide accurate density values, the I(Q) corrections need to be optimal, as G(r) shape is entirely dependent on I(Q) corrections and on the shape of S(Q) dataset towards the high Q’s; the latter will affect the local minimum yielding the number density of the low r’s of the G(r).

The ability of the current methodology to provide an accurate density estimate is intrinsically dependent on the quality of the diffraction dataset. This quality is partially dependent on the attainable Q_max_ which, by extension, affects the low r region through Eq. (1) and the ‘usable’ G(r) range - i.e. the part which is physically meaningful and which is most likely to contain the most negative value for the ratio G(r)/(4πr). We note here that as r_min_ = 2π/Q_max_ it follows that G(r) does not have any physical significance below r_min_, because Q_max_ is the resolution limit of the diffraction method; hence the lower limit of the usable G(r) range is inherently set by r_min_. As the G(r) curves towards increasingly positive values with increasing r, after the first coordination peak (also see reference [8] and references therein), it is justified to consider the position of the first coordination peak as a natural upper limit of the usable G(r) range; as a reminder, the usable G(r) range is comprised of the most negative G(r)/(4πr) values, which are systematically located between r_min_ and the first coordination peak for all amorphous spectra, with no exceptions.

## Method validation

In order to exemplify the use of Eq. (4), here we determine the number density of a mixed Fe-Si-Al-Ca-Mg-Cr-Cu-Ni oxide system in the vitreous state, which we originally discussed elsewhere^7^. For completeness, we note that the sample was aerodynamically levitated by a compressed air flow and melted to 1500 °C by a 100 W CO_2_ laser source. While in flight, the laser beam was switched off, leading to rapid quenching of the sample by the air stream, producing a glassy near-spherical particle with a density of up to 0.088 atoms/Å^3^ (3.54 g/cm^3^) based on a CCD measurement of the particle’s diameter. During levitation, high-energy XRD intensities were collected at a photon wavelength of 0.20194 Å (61.39 keV) yielding a Q range of up to 21 Å^−1^ at low scattering angles (up to 40° 2θ).

**Fig. 1:**
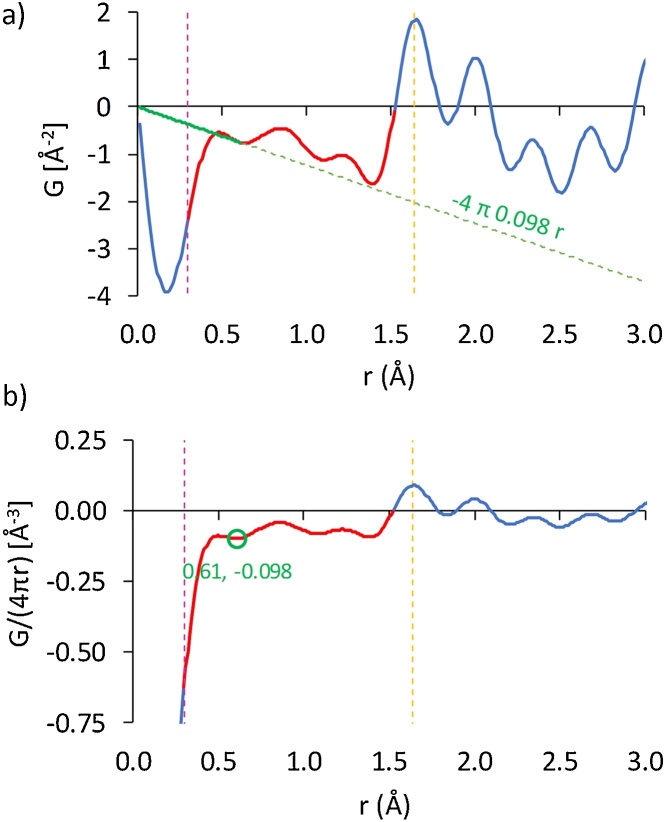
a) G(r) and b) G(r)/(4πr) profiles of the glass sample. In both plots, r_min_ (vertical dashed green line) is equal to 0.3 Å, corresponding to a Q_max_ of 21 Å^−1^ and the first coordination peak (vertical dashed yellow line) is located at 1.65 Å (corresponding to the Si—O bond), as established in our precursor work [6]. Usable spectra are shown in red, superimposed on both profiles. Application of Eq. (4) yielded a number density of 0.098 atoms/Å^3^ based on the local minimum at 0.61 Å, as marked by the green circle in Fig. 1b. Use of this number density resulted in the baseline fit designated by the green line in Fig. 1a. (For interpretation of the references to colour in this figure legend, the reader is referred to the web version of this article).

The G(r) was extracted from I(Q) based on Eq. (1) (the diffraction dataset was Fourier-transformed via IGOR PRO™ running a custom subroutine [7], in the absence of window functions in order to avoid spurious effects). The calculation of amorphous number density based on G(r) is implemented in the spreadsheet included as supplementary material. The procedure first involves inputting the G(r) vs. r dataset (spreadsheet cells downwards from B9 and A9 respectively) and the determination of r_min_ (see spreadsheet cell H3 and purple dashed line in Fig. 1a and b) via Q_max_ (spreadsheet cell H1), followed by identification of the usable G(r) range, i.e. the part of the spectrum lying from r_min_ up to some interatomic distance for which G(r) intercepts the x-axis. Here, we set this distance equal to the position of the first coordination peak (see spreadsheet cell H2 and yellow dashed line in Fig. 1a and b). The usable part of G(r) is designated by a red line superimposed on the PDF profile in Fig. 1a (spreadsheet column C). We then apply Eq. (4) to the usable G(r) range by first dividing G(r) by 4πr (spreadsheet column D) and considering only the negative part of the G(r)/(4πr) range (spreadsheet column F and spectrum designated by the red line in Fig. 1b) for which we proceed to identify all of its local minima. This is done by first calculating the tangent for all pairs of consecutive points on the G(r)/(4πr) plot (spreadsheet column G) and then identifying a tangent sign reversal for each of these pairs, the sign reversal flagging the local minimum (spreadsheet column H). We then store the position, r, as well as the G(r)/(4πr) value for each of the local minima identified. On the basis of Eq. (4), the local minimum with the most negative value, r_d_, is selected as the closest approximation to the material’s number density, ρ_0_, (point designated by the green circle in Fig. 1b and its absolute value in spreadsheet cell H6). In order to visualize the PDF baseline of the glass sample, we may then draw the -4πρ_0_r term between r = 0 and r_d_ (see green solid line in Fig. 1a) and extend it towards high r values (dashed green line in Fig. 1a), the baseline touching the local minimum prior to the first coordination shell, which appears to be a plausible fit to the G(r) profile. In this example, the calculated ρ_0_ compared favorably against the experimentally established number density [6] to within 15.3%.
